# Corneal Endothelial Changes After Phacoemulsification Using the Eight-Chop Technique in Diabetic Eyes

**DOI:** 10.3390/jpm15050209

**Published:** 2025-05-20

**Authors:** Tsuyoshi Sato

**Affiliations:** Department of Ophthalmology, Sato Eye Clinic, 3-3 Nemoto, Matsudo-shi 271-0077, Chiba-ken, Japan; perfect-eightchop@sato-ganka.com

**Keywords:** cataract surgery, corneal endothelial cell, diabetes mellitus, eight-chop technique, phacoemulsification

## Abstract

**Background/Objectives**: To analyze corneal endothelial changes and intraocular pressure (IOP) after phacoemulsification combined with the eight-chop technique and intraoperative parameters in patients with diabetes mellitus. **Methods**: The eyes of patients with cataracts who underwent phacoemulsification were analyzed in this study. Based on their hemoglobin A1c levels, patients were divided into two groups. The eight-chop technique was used for cataract surgery. The operative time, the phaco time, the aspiration time, the cumulative energy dissipated, and the volume of fluid used were determined. Best corrected visual acuity, IOP, corneal endothelial cell density (CECD), central corneal thickness (CCT), coefficient of variation (CV), and percentage of hexagonal cells (PHC) were recorded before and after surgery. **Results**: Overall, 181 eyes of 138 patients with cataracts were evaluated. In the diabetes group, the CECD loss rates were 5.1%, 3.9%, and 2.1% at 7 weeks, 19 weeks, and 1 year postoperatively, respectively. In the control group, the CECD loss rates were 2.8%, 2.6%, and 1.2% at 7 weeks, 19 weeks, and 1 year postoperatively, respectively. Significant differences in the percentage decrease in CECD were observed between the two groups at 7 and 19 weeks postoperatively. Significant differences in the CV and PHC were observed preoperatively and postoperatively between the diabetes and control groups (*p* < 0.01 or *p* = 0.01, 0.02). Significant differences were also observed between CV and PHC preoperatively, at 19 weeks, and 1 year postoperatively in the diabetes and control groups (*p* < 0.01). At 1 year postoperatively, IOP reduction rates were 8.0% and 11.2% in the diabetes and control groups, respectively. **Conclusions**: CECD loss was minimal with the eight-chop technique; however, the diabetes group showed a higher percentage decrease than the control group up to 19 weeks postoperatively. In addition, although IOP decreased in both groups after surgery, the percentage decrease was significantly different at 1 year postoperatively. This study suggests that the corneal endothelial cells of diabetic eyes may be more fragile than those of normal eyes and that the long-term postoperative IOP-lowering effect may be attenuated. These findings will contribute to advances in personalized treatment strategies for patients with diabetes.

## 1. Introduction

Diabetes causes high rates of cataracts and other eye complications [1,2]. In patients with diabetes, high blood sugar is associated with an increase in the accumulation of TGF-β-induced proteins and the formation of advanced glycation end products in the corneal endothelial cells [3]. Components of the extracellular matrix, such as vitronectin and fibronectin, are markedly increased along the Descemet membrane–stromal interface [3]. Furthermore, the activity of Na^+^/K^+^-ATPase, which plays an important role in the maintenance of the cornea, is reduced. Therefore, corneal endothelial cells in patients with diabetes are structurally and functionally impaired [4,5].

Patients with diabetes have higher intraocular pressure (IOP) than healthy participants [6] and are more likely to develop glaucoma than normal individuals [6,7]. Therefore, it is important to investigate changes in IOP after cataract surgery in patients with diabetes who have a high incidence of glaucoma [6,7]. However, many studies have shown that IOP decreases after cataract surgery in patients with and without diabetes, though the results varied [8,9]. Based on the above, changes in corneal endothelial cell density (CECD) and IOP after cataract surgery are important indicators in patients with diabetes.

Advances in phacoemulsification technology have improved the safety and efficacy of cataract surgery; nevertheless, cataract surgery remains a leading cause of CECD loss, and challenges related to corneal endothelial cell protection remain [10]. However, results regarding the reduction in CECD after surgery are inconsistent [11,12,13], and the phacoemulsification techniques used in surgery are mixed. In addition, intraoperative measurement of parameters is an important indicator for analysis of the effects of surgery; however, only a few studies have investigated the relationship between these parameters and postoperative CECD loss. Although cataract surgery alone does not cause corneal endothelial cell dysfunction, the CECD loss after cataract surgery needs to be minimized, considering the effects of potential complications such as glaucoma and vitreous surgery.

The eight-chop technique is a method of dividing the lens nucleus into eight sections before performing phacoemulsification. It is particularly effective in hard nucleus cataracts compared to other techniques. Within a stable anterior chamber filled using an ophthalmic viscoelastic device, the lens nucleus is safely divided using the eight-chopper. With its sharp tip, the eight-chopper can divide almost the entire lens nucleus. This technique improves the efficiency of lens nucleus processing, reduces surgical time, cumulative dissipated energy (CDE), and fluid volume, while minimizing corneal endothelial cell loss [14,15,16]. Furthermore, the CECD loss after cataract surgery is 0.9–1.0%, and IOP also decreases by 12–18% if the nucleus is not hard [14]. From the perspective of corneal endothelial cell protection and IOP-lowering effects, the eight-chop technique is an ideal surgical method.

The purpose of this study was to measure changes in corneal endothelial cells, IOP, and intraoperative parameters using the eight-chop technique in patients with diabetes.

## 2. Materials and Methods

### 2.1. Ethical Considerations

The protocol was approved by the institutional review board and adhered to the tenets of the Declaration of Helsinki. After explaining the nature and possible consequences of the study, informed consent was obtained from each patient to participate in the study.

### 2.2. Study Population

This study enrolled the eyes of patients with cataract who underwent phacoemulsification and posterior chamber intraocular lens (IOL) implantation between June 2019 and July 2022 and visited the Sato Eye clinic (Matsudo-shi, Chiba-ken, Japan). Exclusion criteria were corneal disease or opacity, uveitis, diabetic retinopathy, poorly dilated pupils (<5.0 mm), white cataracts, preoperative CECD < 2000 cells/mm^2^, severely weak zonules, and past ocular trauma or surgery.

### 2.3. Preoperative Assessment

All patients underwent slit-lamp and retinal biomicroscopy, best corrected visual acuity (BCVA), and IOP testing preoperatively. The patients were diagnosed with diabetes based on the Hemoglobin A1c level within 6 months prior to surgery. The CECD (cells/mm^2^), central corneal thickness (CCT), coefficient of variation (CV), and percentage of hexagonal cells (PHCs) were analyzed with a non-contact specular microscope (EM-3000; Topcon Corporation, Tokyo, Japan). The hardness of the nucleus was graded according to the Emery classification [17]. All patients were treated by the same surgeon experienced in the eight-chop technique with the phacoemulsification system (Centurion^®^; Alcon Laboratories, Inc., Irvine, CA, USA).

### 2.4. Surgical Technique

In all cases, a 3.0 mm steel keratome was used to create a temporal clear corneal incision. After the injection of sodium hyaluronate into the anterior chamber, a 6.2–6.5 mm continuous curvilinear capsulorrhexis was constructed with capsule forceps. Hydrodissection was achieved with a 27-G cannula. The lens nucleus was divided into eight segments using an eight-chopper I or II in the grade II and III groups. At the depth of the iris plane, the eight segments were phacoemulsified and aspirated. The capsular bag was aspirated with an irrigation/aspiration tip to remove the cortical materials. The ophthalmic viscosurgical device was injected, and a foldable IOL (Acrysof^®^ MN60AC; Alcon Laboratories, Inc., Fort Worth, TX, USA), with polymethyl methacrylate haptics, was placed into the capsular bag via injection. Consequently, the ophthalmic viscosurgical device was removed. Postoperatively, the anterior chamber was exchanged with a balanced salt solution containing moxifloxacin (0.5 mg/mL).

Intraoperative outcome measures were operative time in minutes, phaco time in seconds, aspiration time in seconds, cumulative dissipated energy (CDE), volume of fluid used in milliliters, and intraoperative complication incidence. The operative time was calculated from the beginning of the corneal incision to the end of aspiration of the ophthalmic viscosurgical device. All surgeries were captured on camera (MKC-704KHD, Ikegami Tsushinki co., Ltd., Tokyo, Japan), and the video images were saved on a hard disk.

### 2.5. Data Collection and Statistical Analysis

Patients were observed at postoperative days 1 and 2, weeks 1, 3, 7, and 19, and year 1. Postoperative outcome measures included BCVA, IOP, CCT, CV, PHC, and CECD at 7 weeks, 19 weeks, and 1 year postoperatively.

Mann–Whitney U tests were used for statistical analyses to compare results between the diabetes and control groups. A paired *t*-test was used to compare the preoperative BCVA, IOP, CV, PHC, CCT, and CECD values with the values at each postoperative time point. The level of statistical significance was set at *p* < 0.05. Chi-squared test was used to determine whether there were differences between the diabetes and control groups by sex.

## 3. Results

### 3.1. Characteristics of the Participants

This study investigated 181 eyes of 138 patients with cataract undergoing phacoemulsification and IOL implantation. The characteristics of the patients and the intraoperative parameters are presented in Table 1. No significant difference was observed between the diabetes and control groups in terms of mean age (*p* = 0.71) and sex (*p* = 0.12). In addition, no significant differences were observed between the diabetes and control groups in terms of operative time, phaco time, aspiration time, CDE, and volume of fluid used (*p* = 0.14, 0.15, 0.83, 0.14, and 0.65, respectively).

### 3.2. Changes in CECD

Preoperative and postoperative changes in CECD are shown in Table 2. There was no significant difference in CECD between diabetes and control groups preoperatively and at 7 weeks, 19 weeks, and 1 year postoperatively (*p* = 0.61, 0.24, 0.73, and 0.99, respectively). In the diabetes and control groups, significant differences were observed between preoperative and 7-week postoperative CECD, preoperative and 19-week postoperative CECD, and preoperative and 1-year postoperative CECD (all *p* < 0.01). Regarding the percentage decrease in CECD, significant differences were observed between the two groups at 7 and 19 weeks postoperatively; however, no significant differences were observed at 1 year postoperatively (*p* < 0.01, *p* = 0.03, 0.10, respectively).

### 3.3. Changes in CCT, CV, and PHC

Preoperative and postoperative changes in the CCT, CV, and PHC are shown in Table 3. Between the diabetes and control groups, there were no significant differences in the CCT preoperatively and at 7 weeks, 19 weeks, and 1 year postoperatively (*p* = 0.59, 0.09, 0.26, and 0.24, respectively). There were significant differences in the CV preoperatively and at 7 and 19 weeks and 1 year postoperatively between the diabetes and control groups (*p* < 0.01 or *p* = 0.01). Between the diabetes and control groups, significant differences were observed in the PHC preoperatively and at 7 weeks, 19 weeks, and 1 year postoperatively (*p* < 0.01 or *p* = 0.02). There were no significant differences between the CCT preoperatively and that at 1 year postoperatively in the diabetes group (*p* = 0.38); however, there were significant differences between the CCT preoperatively and that at 1 year postoperatively in the control group (*p* < 0.01). In addition, CV and PHC showed significant differences between preoperative and 1-year postoperative values in both groups (all *p* < 0.01).

### 3.4. Changes in IOP

Changes in IOP are shown in Table 4. There were significant differences in IOP preoperatively; however, there were no significant differences in IOP at 7 weeks, 19 weeks, and 1 year postoperatively between the diabetes and control groups (*p* = 0.01, 0.48, 0.19, and 0.36, respectively). There were significant differences between IOP preoperatively and that at 7 weeks, 19 weeks, and 1 year postoperatively in the diabetes and control groups (all *p* < 0.01). Regarding the percentage decrease in IOP, no significant differences were observed between the two groups at 7 and 19 weeks postoperatively; however, significant differences were observed at 1 year postoperatively (*p* = 0.07, 0.26, and 0.02, respectively).

### 3.5. Changes in BCVA over the Course of Time

Changes in BCVA are shown in Table 5. There were significant differences in the preoperative BCVA between the diabetes and control groups (*p* = 0.02). Moreover, there were significant differences in the BCVA at 7 weeks, 19 weeks, and 1 year postoperatively between the diabetes and control groups (all *p* < 0.01). In the diabetes and control groups, there were significant differences between the BCVA preoperatively and that at 7 weeks postoperatively; between the BCVA preoperatively and that at 19 weeks postoperatively; and between the BCVA preoperatively and that at 1 year postoperatively (all *p* < 0.01).

### 3.6. Complications

There were no intraoperative complications or capsulorrhexis tears in the diabetes or control groups.

## 4. Discussion

Previous studies have reported 10.2–18.5% and 5.0–18.4% decreases in the CECD following cataract surgery in the first few postoperative months in patients with and without diabetes [10,18,19]. In this study, however, the decrease was only 5.1%, 3.9%, and 2.1% at 7 weeks, 19 weeks, and 1 year postoperatively, respectively, in the diabetes group and 2.8%, 2.6%, and 1.2% at 7 weeks, 19 weeks, and 1 year postoperatively, respectively, in the control group. Diabetes increases the risk of damage and dysfunction of the corneal endothelium following surgical stress [20]. Compared with that of patients without diabetes, those with diabetes show differences in greater CECD loss following cataract surgery [18,21,22]. However, although the percentage decrease in postoperative CECD was significantly different between the diabetes group and control groups by 19 weeks postoperatively, no significant difference in CECD was observed. The low surgical invasiveness of the eight-chop technique may have prevented the fragility of the corneal endothelial cell function of patients with diabetes from causing a difference in the CECD loss. CECD loss during phacoemulsification is inevitable. This is primarily due to the confined space (anterior chamber), where the surgery is performed. The corneal endothelium comes into direct contact with the instruments several times during the procedure, making it susceptible to damage from the high ultrasound energy of the phaco tip [23]. Despite the potential benefits of phacoemulsification, the CECD after phacoemulsification remains a major concern. CECD assessment is critical for comparing different techniques because it represents the true summary of intraocular insult during surgery [24]. Our results suggest that the eight-chop technique may be advantageous in minimizing surgical involvement of intraocular tissues, including the trabecular meshwork and Schlemm’s canal. Modern cataract surgery aims not only to improve vision but also to minimize the damage to corneal endothelial cells, especially in patients with cataracts and diabetes. This study revealed that, in patients with diabetes, the CECD loss after cataract surgery is greater than that in normal patients; however, this loss is minimal. Moreover, if the eight-chop technique is used, even in patients with diabetes, the rate of CECD reduction 1 year post-surgery is lower than the average annual rate of decrease over 10 years post-surgery in normal patients, which is at 6.7% [25]. Furthermore, the preoperative CECD in patients with diabetes was lower than that in the healthy population [18,19]; conversely, Yang et al. [22] reported that there was no difference and no agreement. In this study, no significant differences were observed in the CECD preoperatively between the diabetes and control groups.

In both the diabetes and control groups, the results of this study indicated that the eight-chop technique exhibited an operative time of 4.63–4.91 min. This time frame was found to be significantly less than that of other techniques, which ranged from 10 to 19 min [10,26]. Additionally, the phaco time and CDE were reduced, and the volume of fluid used was decreased from one-third to one-fourth of that used in other techniques [10,27]. The eight-chop technique involves the mechanical division of the nucleus into eight pieces preceding phacoemulsification. This results in the reduced use of ultrasonic oscillation energy and an efficient removal of the fragmented nucleus, which can lead to a drastic reduction in the phaco time and CDE. The concept of the eight-chop technique may be analogous to femtosecond laser-assisted cataract surgery in that both procedures involve the division of the lens nucleus without the utilization of ultrasonic oscillation energy.

CCT is used as a marker of corneal endothelial function [21]. In this study, preoperative and postoperative CCT were not significantly different between the diabetes and control groups. Nevertheless, significant differences were observed between the CCT preoperatively and that at 7 weeks postoperatively and between the CCT preoperatively and that at 19 weeks postoperatively in the diabetes group, whereas no significant differences were observed in the control group. This result shows that there is a possibility of corneal endothelial cell dysfunction in the diabetes group at 19 weeks postoperatively compared to that in the control group. The CV is an indicator of the uniformity in the size of endothelial cells, which implies the repair and healing mechanisms of the endothelium after damage. Previous studies have demonstrated significant and non-significant differences in CV changes between patients with and without diabetes after cataract surgery [18,22]. In this study, the CV in the diabetes group was higher than that in the control group preoperatively, and the CVs decreased significantly in both groups at 1 year postoperatively; moreover, there was no significant difference between the two groups. Hexagonality indicates the variability in hexagonal cell shape, such as the CV; moreover, it represents the healing response after damage [21]. Previous studies have demonstrated significant and non-significant differences in PHC changes between patients with and without diabetes after cataract surgery [18,22]. In this study, the PHC in the diabetes group was lower than that in the control group preoperatively; the PHCs increased significantly in both groups at 1 year postoperatively, with no significant difference between the two groups. These results of CV and PHC suggest that the repair and healing mechanisms of the endothelium may be reduced in the diabetes group preoperatively, although their activity may have increased in both groups postoperatively because of the low surgical invasiveness of the eight-chop technique.

Many investigators have reported IOP reduction after phacoemulsification, cataract extraction, and IOL implantation in patients with cataract [28,29]. Previous reports have demonstrated IOP reductions of 4–10% [30,31]. The change in postoperative IOP is proportional to the change in preoperative IOP. On the other hand, Poly et al. [31] reported an increase in IOP in the primary open-angle glaucoma (POAG) and normal groups at 1 year in comparison with the preoperative levels. Therefore, there are conflicting perspectives on IOP reduction after phacoemulsification cataract surgery. The study showed an IOP reduction rate of 8.0% and 11.2% for the diabetes and control groups, respectively, at 1 year postoperatively, which was higher than previously reported data in both groups. The greater IOP reduction could be attributed to the superiority of the eight-chop technique over other techniques in decreasing surgical involvement of the intraocular tissues.

Trabecular meshwork cells, which are exposed to the same ultrasonic energy and turbulent currents as corneal endothelial cells during cataract surgery, are expected to decrease in number if the corneal endothelial cells decrease more severely. A decrease in the trabecular meshwork cells is associated with POAG and elevated IOP. POAG eyes contained overall fewer trabecular meshwork cells than normal eyes, indicating an average cell loss of 17.7% in glaucoma eyes [32]. Furthermore, trabecular meshwork cellularity was highly correlated with the maximum recorded IOP [32], supporting the notion that reduced trabecular meshwork cell density hampers the ability of the tissue to regulate IOP [32]. Therefore, increasing CECD loss may lead to trabecular meshwork cell loss, resulting in increased IOP. In the eight-chop technique, the low volume of fluid used suggests that the ultrasonic and irrigation/aspiration tips are employed for a shorter period, which may exert a reduced impact on the trabecular meshwork and Schlemm’s canal cells, including corneal endothelial cells. Therefore, we speculate that the eight-chop technique may result in less trabecular meshwork cell loss, and as a result, normal trabecular meshwork function may be maintained, leading to long-term IOP reduction.

It is important to note that this study is not without limitations. First, the initial observation is derived from the absence of outcomes with the prechop, phaco-chop, or divide-and-conquer techniques. This should be taken into full consideration when assessing the present results. However, numerous other studies have been carried out employing the phaco-chop and divide-and-conquer techniques, and it is our belief that the present results can be assessed by comparing them with our results. Second, the correlation between intraoperative parameters, postoperative outcomes, blood glucose levels, duration of diabetes, and type of diabetes was not investigated. Furthermore, as cases with diabetic retinopathy were excluded, it is unknown how the CECD changes in patients with severe diabetes after cataract surgery.

The prechop technique has the excellent feature of being able to reduce the use of ultrasonic oscillation energy and the surgical invasion of intraocular tissue by mechanically dividing the crystalline lens into four segments before phacoemulsification [33]. This excellent feature is the same as the advantages of femtosecond laser-assisted cataract surgery. However, it is far superior to femtosecond laser-assisted cataract surgery in terms of medical economics. Although the prechop technique has excellent features, it is rarely used as a surgical technique worldwide [14,34] because the prechopper may not be easy to operate. However, the eight-chop technique, which is an improvement on the prechop technique and has improved usability [14,15]. Furthermore, by using the Lance-chopper, it is possible to perform phacoemulsification surgery on patients with hard nucleus cataracts or weak zonules [14]

When analyzing changes in IOP and CECD after phacoemulsification cataract surgery, phaco time, aspiration time, CDE, volume of fluid used, and operative time should be carefully evaluated. However, to the best of our knowledge, few studies have evaluated the effects of phacoemulsification cataract surgery on IOP and CECD. To reduce postoperative CECD loss and maintain IOP reduction, surgical invasion of intraocular tissues should be analyzed using intraoperative parameters, and a phacoemulsification technique should be selected to minimize surgical invasion.

## 5. Conclusions

The eight-chop technique is a cataract surgery with a shorter operative time, less surgical invasion of intraocular tissues, and less fluid volume used than those in previous techniques. The CECD loss in the present study was minimal compared with that reported in previous studies. The changes in the CV and PHC suggest that the repair and healing mechanisms of the endothelium may have increased postoperatively because of the low surgical invasiveness of the eight-chop technique. The IOP reduction could be explained by the superiority of the eight-chop technique in minimizing surgical involvement of the intraocular tissues. Future research on the loss of CECD in patients with diabetes after cataract surgery will advance personalized treatment strategies and lead to improved management and treatment of cataract.

## Figures and Tables

**Table 1:** Preoperative characteristics and intraoperative parameters.

Characteristics/Parameters	Diabetes Group	Control Group	*p*-Value
Number of eyes	94	87	
Age (years)	74.6 ± 6.8	74.3 ± 5.5	0.71 ^a^
Sex Male	43 (46%)	30 (34%)	0.12 ^b^
Female	51 (54%)	57 (66%)	
Operative time (min)	4.63 ± 1.24	4.91 ± 1.38	0.14 ^a^
Phaco time (s)	15.5 ± 6.0	14.2 ±6.3	0.15 ^a^
Aspiration time (s)	68.1 ± 16.7	67.6 ± 20.4	0.83 ^a^
CDE	6.45 ± 2.30	5.91 ± 2.61	0.14 ^a^
Volume of fluid used (mL)	27.0 ± 7.7	26.5 ± 8.1	0.65 ^a^

Values are expressed as mean ± standard deviation or as percentages, unless otherwise noted. ^a^ No significant differences were found between the groups using the Mann–Whitney U test. ^b^ No significant differences were found between the groups using the Chi-square test. CDE, cumulative dissipated energy.

**Table 2:** Pre- and postoperative CECD values.

	Mean CECD ± SD (% Decrease)		
Time Period	Diabetes Group (*n* = 94)	Control Group (*n* = 87)	*p*-Value
Preoperatively	2670 ± 294	2652 ± 211	0.60 ^a^
7 weeks postoperatively	2533 ± 272 ^b^	2576 ± 207 ^b^	0.23 ^a^
% Decrease	5.1 ± 4.7	2.8 ± 2.5	<0.01 ^c^
19 weeks postoperatively	2570 ± 294 ^b^	2583 ± 221 ^b^	0.72 ^a^
% Decrease	3.9 ± 4.8	2.6 ± 2.5	0.03 ^c^
1 year postoperatively	2620 ± 306 ^b^	2620 ± 214 ^b^	0.99 ^a^
% Decrease	2.1 ± 4.5	1.2 ± 1.9	0.10 ^a^

Values represent mean ± standard deviation. ^a^ No significant differences were found between the groups using the Mann–Whitney U test. ^b^ Significant differences were found between the preoperative and respective time values using a paired *t*-test. ^c^ Significant differences were found between the groups using the Mann–Whitney U test. CECD, corneal endothelial cell density; SD, standard deviation.

**Table 3:** Pre- and postoperative endothelial CCT, CV, and PHC.

Time Period	Diabetes Group (*n* = 94)	Control Group (*n* = 87)	*p*-Value
CCT	Mean ± SD		
Preoperatively	538 ± 34.0	536 ± 34.1	0.59 ^a^
7 weeks postoperatively	547 ± 36.2 ^c^	537 ± 34.0 ^d^	0.09 ^a^
19 weeks postoperatively	542 ± 33.3 ^c^	536 ± 32.7 ^d^	0.26 ^a^
1 year postoperatively	537 ± 35.0 ^d^	531 ± 33.6 ^c^	0.24 ^a^
CV	Mean ± SD		
Preoperatively	42.3 ± 5.5	39.8 ± 6.9	<0.01 ^b^
7 weeks postoperatively	41.3 ± 5.6 ^d^	39.1 ± 5.2 ^d^	<0.01 ^b^
19 weeks postoperatively	39.3 ± 6.0 ^c^	36.4 ± 4.9 ^c^	<0.01 ^b^
1 year postoperatively	37.6 ± 5.0 ^c^	35.7 ± 4.9 ^c^	0.01 ^b^
PHC	Mean ± SD		
Preoperatively	39.7 ± 6.9	44.7 ± 5.6	<0.01 ^b^
7 weeks postoperatively	41.0 ± 6.5 ^d^	45.5 ± 6.7 ^d^	<0.01 ^b^
19 weeks postoperatively	44.5 ± 7.1 ^c^	48.1 ± 6.2 ^c^	<0.01 ^b^
1 year postoperatively	46.4 ± 6.7 ^c^	48.8 ± 7.0 ^c^	0.02 ^b^

Values represent mean ± standard deviation. ^a^ No significant differences between the groups using the Mann–Whitney U test. ^b^ Significant differences between the groups using the Mann–Whitney U test. ^c^ Significant differences between the preoperative and respective time values using a paired *t*-test. ^d^ No significant differences between the preoperative and respective time values using a paired *t*-test. CCT, central corneal thickness; CV, coefficient of variation; PHC, percentage of hexagonal cells; SD, standard deviation.

**Table 4:** Mean IOP (mmHg) and mean decrease (%) in the IOP (mmHg) over the course of time.

	Mean IOP ± SD (% Decrease)		
Time Period	Diabetes Group (*n* = 94)	Control Group (*n* = 87)	*p*-Value
Preoperatively	13.7 ± 2.1	14.5 ± 2.0	0.01 ^a^
7 weeks postoperatively	11.9 ± 2.3 ^b^	12.1 ± 1.8 ^b^	0.48 ^c^
% Decrease	13.2 ± 11.6	16.1 ± 9.1	0.07 ^c^
19 weeks postoperatively	12.3 ± 2.2 ^b^	12.6 ± 1.8 ^b^	0.19 ^c^
% Decrease	10.7 ± 10.2	12.4 ± 10.0	0.26 ^c^
1 year postoperatively	12.6 ± 2.1 ^b^	12.9 ± 1.9 ^b^	0.36 ^c^
% Decrease	8.0 ± 10.9	11.2 ± 7.4	0.02 ^a^

Values represent mean ± standard deviation. ^a^ Significant differences between the groups using the Mann–Whitney U test. ^b^ Significant differences between the preoperative and respective time values using a paired *t*-test. ^c^ No significant differences between the groups using the Mann–Whitney U test. IOP, intraocular pressure; SD, standard deviation.

**Table 5:** Changes in best-corrected visual acuity over the course of time.

	Best-Corrected Visual Acuity		
Time Period	Diabetes Group (*n* = 94)	Control Group (*n* = 87)	*p*-Value
Preoperatively	0.031 ± 0.030	0.074 ± 0.011	0.02 ^a^
7 weeks postoperatively	−0.040 ± 0.0049 ^c^	−0.065 ± 0.00095 ^c^	<0.01 ^b^
19 weeks postoperatively	−0.037 ± 0.0038 ^c^	−0.0664 ± 0.00086 ^c^	<0.01 ^b^
1 year postoperatively	−0.039 ± 0.0042 ^c^	−0.064 ± 0.0010 ^c^	<0.01 ^b^

Values represent mean ± standard deviation. ^a^ No significant differences between the groups using the Mann–Whitney U test. ^b^ Significant differences between the groups using the Mann–Whitney U test. ^c^ Significant differences between the preoperative and respective time values using a paired *t*-test.

## Data Availability

Owing to privacy and ethical restrictions, the data presented in this study are available upon request to the corresponding author.

## Abbreviations

The following abbreviations are used in this manuscript:

IOP	Intraocular pressure
CECD	Corneal endothelial cell density
CCT	Central corneal thickness
CV	Coefficient of variation
PHC	Percentage of hexagonal cells
IOL	Intraocular lens
BCVA	Best-corrected visual acuity
CDE	Cumulative dissipated energy
SD	Standard deviation
POAG	Primary open-angle glaucoma
